# Why do health technology assessment coverage recommendations for the same drugs differ across settings? Applying a mixed methods framework to systematically compare orphan drug decisions in four European countries

**DOI:** 10.1007/s10198-016-0823-0

**Published:** 2016-08-18

**Authors:** Elena Nicod

**Affiliations:** Department of Social Policy, LSE Health and Social Care, London School of Economics and Political Science, Houghton Street, London, WC2A 2AE UK

**Keywords:** Health technology assessment, Orphan drugs, France, England, Scotland, Sweden, Mixed methods research, Thematic analysis

## Abstract

**Purpose:**

Health technology assessment (HTA) coverage recommendations differ across countries for the same drugs. Unlike previous studies, this study adopts a mixed methods research design to investigate, in a systematic manner, these differences.

**Methods:**

HTA recommendations for ten orphan drugs appraised in England (NICE), Scotland (SMC), Sweden (TLV) and France (HAS) (*N* = 35) were compared using a validated methodological framework that breaks down these complex decision processes into stages facilitating their understanding, analysis and comparison, namely: (1) the clinical/cost-effectiveness evidence, (2) its interpretation (e.g. part of the deliberative process) and (3) influence on the final decision. This allowed qualitative and quantitative identification of the criteria driving recommendations and highlighted cross-country differences.

**Results:**

Six out of ten drugs received diverging HTA recommendations. Reasons for cross-country differences included heterogeneity in the evidence appraised, in the interpretation of the same evidence, and in the different ways of dealing with the same uncertainty. These may have been influenced by agency-specific evidentiary, risk and value preferences, or stakeholder input. “Other considerations” (e.g. severity, orphan status) and other decision modulators (e.g. patient access schemes, lower discount rates, restrictions, re-assessments) also rendered uncertainty and cost-effectiveness estimates more acceptable. The different HTA approaches (clinical versus cost-effectiveness) and ways identified of dealing with orphan drug particularities also had implications on the final decisions.

**Conclusions:**

This research contributes to better understanding the drivers of these complex decisions and why countries make different decisions. It also contributed to identifying those factors beyond the standard clinical and cost-effectiveness tools used in HTA, and their role in shaping these decisions.

## Introduction

Providing equal access to affordable medicines across countries is high on the political agenda in many OECD countries including those in the European Union [1]. In reality, this is far from being achieved even in countries with similar or comparable policies, rules or priorities. In countries using health technology assessment (HTA) to inform resource allocation decisions, important disparities across countries in their HTA coverage recommendations for the same drug are often reported [2–4]. These divergences may relate to contextual differences such as the objectives adopted, where it might be a pharmacoepidemiological study in one country and a systematic review of all aspects of using a technology in another [5]. Equally, there may be different willingness-to-pay thresholds affecting the extent to which an HTA outcome is acceptable [6, 7]. Differences may also be due to controversies over the HTA process itself, including questions about the most appropriate methodological approach to undertaking HTA [8, 9], the application of HTA in each setting, whether the measures used fully capture the effects and costs from taking the treatment [10–12], what levels of evidence are acceptable [13, 14], how to deal with uncertainty [15], or to what extent “other considerations”, e.g. disease and treatment characteristics, were consistent across decisions [16].

This problem, together with its implications, has been identified and possible explanations examined. Eight studies compared HTA drug coverage recommendations across countries and highlighted the extent of these differences [2, 3, 17–22]. Their research designs were in the form of retrospective descriptive or cohort analyses, and countries compared included Canada, Australia, England, Scotland, France and New Zealand. The reasons for cross-national differences were also explored, but with varying levels of thoroughness. Morgan and colleagues focused on the transparency and rigour of the processes rather than on case-specific reasons for diverging recommendations [17]. Three other studies investigated the reasons for these differences [2, 3, 18], but relied on few cases or potential reasons. First, they did not outline the key determinants or structure of the decision-making explored, where the reasons set forth may not constitute the full picture. Second, issues relating to the clinical and pharmacoeconomic assessments were identified. However, the level of detail provided in their assessments did not differentiate for the type of uncertainty, how they were dealt with and what factors influenced these processes across settings. Third, the methodological approaches used were not sufficiently detailed for these approaches to be transferable. Given that these decision processes are complex and understanding what happened for the same drug in different countries may be challenging, a more systematic, structured and comprehensive approach to identifying and comparing differences would be required. Additionally, understanding how similar scenarios were dealt with across settings may also constitute a way forward to identify limitations in applying HTA and learn from how these were dealt with across settings [4].

Through the application of a validated mixed methods framework [4], the objectives of this study were twofold: to systematically investigate the drivers of HTA recommendations for a sample of orphan drugs in four countries, and to identify the reasons for cross-country differences. The subject matter of the analysis was orphan drugs as they are often cost-ineffective due to the small patient numbers, heterogeneous nature of the conditions they treat, and their often high acquisition price [23–26]. Different studies nevertheless demonstrated that orphan drugs receive the same or a higher level of acceptance compared to other drugs treating more prevalent disease areas [27–29]. Special attention was given to understanding the level of uncertainty characterising orphan drugs, how it was dealt with, and how disease and drug-specific characteristics were accounted for.

## Methods

### Sampling of study countries and drug-indication pairs

Four of the most well-established European HTA bodies were included in the study based on purposive sampling [4], which use clinical or cost-effectiveness as decision-making criteria and for which the reports stating the HTA recommendation and reasons were publicly available. These included the National Institute for Health and Care Excellence (NICE) in England, the Scottish Medicines Consortium (SMC) in Scotland, the Dental and Pharmaceutical Benefits Board (TLV) in Sweden and the Haute Autorité de Santé (HAS) in France. The conventional HTA processes for both orphan and non-orphan indications were examined. The study countries make no differentiation of drugs’ orphan status, with the exception of the SMC and its SMC modifiers. SMC will accept more uncertainty in the health economic case or higher cost/QALYs for orphan drugs. Additional factors, e.g. the SMC modifiers, are considered when assessing the acceptability of uncertainty and high incremental cost-effectiveness ratios (ICERs) [30].

All drug-indication pairs with an orphan designation from the European Medicines Agency (EMA) [31] and appraised by NICE through the Single Technology Appraisal process until December 2012 were included and recorded by their indication, generic name and HTA recommendation. The HTA recommendation was categorised as to list, restrict or reject a drug for coverage. The decision by HAS relies on the drug’s medical benefit (SMR) driving the coverage rate (e.g. 65, 35, 15 %) and the relative improvement in medical benefit (ASMR) providing the price fixing regime applicable, ranging from major to insufficient. Two hundred and sixty-nine technology appraisal reports were published up to December 2012 by NICE, 23 of which received an orphan EMA designation. Excluded were those that underwent the multiple technology appraisal process or were terminated at the time of data collection at NICE (9/23), and those that were appraised by fewer than three of the four study countries (4/23). Those compounds that underwent the abbreviated procedure at SMC were not included since the rationale for the decision was not available. Ten unique orphan drug-indication pairs and a total of 35 country and drug-indication pairs were selected (Table 1). Only five were included by TLV, which appraised mainly outpatient drugs at the time of the study, while many of the study drugs were inpatient [32].

**Table 1:** List of drug-indication pairs included in the study

Generic/brand name	Indication	NICE England	SMC Scotland	TLV Sweden	HAS—France^a^ SMR (coverage) ASMR (pricing)
Eltrombopag REVOLADE	Chronic idiopathic thrombocytopenic purpura	DNL	LWC	LWC	Important (65 %) II (EU)
Romiplostim NPLATE	Chronic idiopathic thrombocytopenic purpura	LWC	LWC	LWC	Important (65 %) II (EU)
Everolimus AFINITOR	Renal cell carcinoma (2nd line, advanced)	DNL	DNL	L	Important (100 %) IV (comp)
Lenalidomide REVLIMID	Multiple myeloma (3rd line)	LWC	LWC	L	Important (65 %) III (EU)
Mifamurtide MEPACT	Osteosarcoma	LWC	L	LWC	Insufficient (0 %) DNL
Azacitidine VIDAZA	Myelodysplastic syndrome	LWC	LWC	NA	Important (65 %) II (EU)
Imatinib GLIVEC	Gastro intestinal stromal tumour (adjuvant, after surgery)	DNL	LWC	NA	Important (100 %) III (EU)
Mannitol dry BRONCHITOL	Cystic fibrosis	LWC	DNL	NA	Weak (15 %) V (comp)
Ofatumumab ARZERRA	Chronic lymphocytic leukemia	DNL	DNL	NA	Moderate (35 %) V (comp)
Trabectedin YONDELIS	Soft tissue sarcoma	LWC	DNL	NA	Important (65 %) V (comp)

*NICE* National Institute for Health and Care Excellence (NICE), *SMC* Scottish Medicines Consortium, *TLV* Dental and Pharmaceutical Benefits Board, *HAS* Haute Autorité de Santé, *L* list, *LWC* list with restrictions, *DNL* do not list, *NA* not applicable, *EU* price negotiation at European price levels, *comp* price set below comparator price

^**a**^The ASMR (Amélioration du Service Médical Rendu) ranks drugs according to their relative improvement in clinical benefit in five levels, from a major innovation (level I) to no improvement (level V). The pricing scheme is determined by the ASMR ranking [e.g. ASMR I–III = price negotiations within European price levels (EU), ASMR IV–V = price set below comparators (comp)]. The SMR (Service Médical Rendu) ranks the drug according to the drug’s clinical benefit in four levels (insufficient, weak, moderate, important) and drives the coverage rate (0, 15, 35, 65 %)

### Study design and methodological framework

**Fig. 1:**
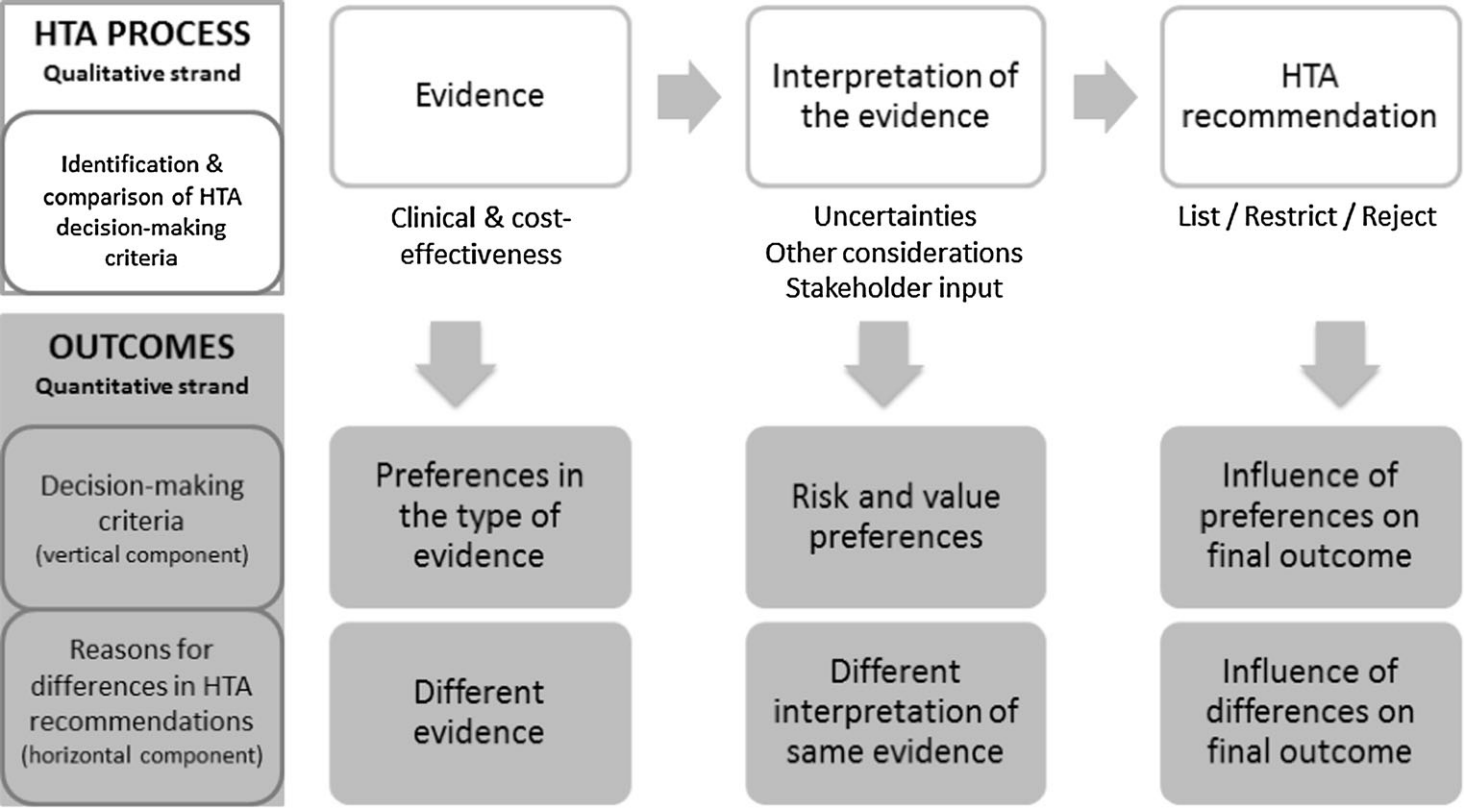
Methodological framework [4] applied to systematically compare HTA decision processes across countries. The HTA process was divided into 3 stages: the evidence appraised (e.g. trial type, clinical and safety endpoints, comparators, economic models), the interpretation of this evidence (e.g. nature of uncertainty, how it was dealt with and the influence of stakeholder input and “other considerations”) and their influence on the final recommendation. Uncertain evidence was defined as evidence considered not fully capturing the effects of a treatment in the intended population by the assessors. “Other considerations” was defined as the non-quantifiable or non-quantified considerations relating to treatment or disease characteristics not captured by routine methods of HTA (e.g. QALY). A number of criteria considered at each stage of the process were qualitatively collected, and quantitatively analysed to determine the criteria driving these decisions (vertical component) and the reasons for differences across countries (horizontal component) [4]

Mixed methods were used to systematically examine the HTA decision processes for individual drugs and countries on the basis of a validated methodological framework. The approach used was an exploratory sequential mixed methods design, where the qualitative strand took priority and preceded the quantitative strand. The framework consisted in a coding manual and case study template [4]. This allowed breaking down of HTA decisions into different stages facilitating their understanding, analysis and comparison in terms of: (a) the clinical and/or cost-effectiveness evidence appraised, (b) the interpretation of this evidence (e.g. as part of the deliberative process) and (c) their influence on the final recommendation (Fig. 1 ) [4].

This multi-level research design allowed for an in-depth analysis of the criteria driving these decision-making processes and of their role in shaping these decision processes in each country, and whether they explained cross-national differences. This research did not aim to generalise findings, but was interested in exploring and elucidating the reasons behind the HTA decisions, which are mainly qualitative in nature [33]. The quantitative strand aimed to complement and enhance the interpretation of the qualitative findings, and to produce more structured data to be used for subsequent analyses.

### Data analysis

Data sources comprised publicly available HTA reports, other official documents (e.g. memos in Sweden) and comments from competent authorities. Although the aims of the HTA reports differ (e.g. advice versus decision), they were assumed to be transparent and reflect the key determinants driving the recommendations [34]. The main results were presented to, and discussed with the HTA bodies. Results were also regularly presented to HTA experts (e.g. Advance-HTA consortium) at various occasions, where feedback was collected. This contributed to ensuring that the interpretation of the decisions made by the researcher was accurate.

Qualitative analysis was conducted in the first stage of the research. On the basis of the framework, all the relevant information at each step of the decision process was identified. This information was compiled into existing case study forms to ensure its completeness and comparability across countries. Thematic analysis was undertaken to code this information in the HTA reports using the NVivo 10 software [35]. Coding was flexible and iterative with new codes being created for all newly identified criteria and included in the coding manual with their definition and coding rule, ensuring that the multiple dimensions of the decision-making process were captured. The HTA reports already coded were re-examined with these new codes, and adjustments were made if necessary. Intra-coding reliability was tested for consistency of coding, and content validity for the representativeness and homogeneity of the information coded within codes [36]. The data collected was exported into excel for analysis using different coding matrix queries.

The qualitative data collected were transformed into quantitative categorical nominal variables by exporting the data into Stata 13 [37]. Thematic matrixes and descriptive statistics were used to determine types and frequencies of variables, their influence on the final recommendation, and how they compared across countries. Correspondence analysis was used to measure agency-specific risk preferences derived from the types of uncertainty, and value preferences derived from the “other considerations” identified [38, 39]. It allowed measurement of the associations between these variables using the chi-squared statistic test of independence and facilitated the understanding of these complex relationships in a bi-dimensional graphical representation [40]. For comparability purposes, TLV was not included in this first part of the analysis but in a secondary analysis relying on the five drugs commonly appraised by all.

Descriptive statistics were used to measure the frequency of agreement across countries in their interpretation of the evidence. Cohen’s kappa scores of cross-country agreement levels were measured to check the robustness of the results obtained by the primary metric, and was done so in a comparable manner given that it focused on each individual concern (uncertainty) raised that was common across settings [41]. Two categories of agreement were measured: (a) the issues raised by each agency about the same evidence, and (b) how the same issues raised by at least two agencies were dealt with across settings. This allowed comparison of observed agreement with agreement expected by chance, ranging from poor (*κ* = 0) to perfect agreement (*κ* = 1), and where negative values of *κ* correspond to cases when agreement was less than that expected by chance [42].

Finally, the analysis also aimed to identify those issues or considerations that relate to the rarity of these conditions, and understand and compare the different approaches to dealing with them across settings.

## Results

Six of the ten study drugs received diverging recommendations, e.g. positive or restricted in some countries and rejected in others (Table 1). Out of the four remaining cases with homogeneous recommendations, romiplostim and lenalidomide were restricted in their indications in some countries and not in others, and ofatumumab was rejected by NICE and SMC and received the lowest ASMR V rating with a moderate SMR rating (30 % reimbursement rate). In only one case (azacitidine) were the recommendations issued really similar. Contrasting trends were also seen, where, for example, mifamurtide received a positive recommendation from NICE and SMC, but was considered insufficient and rejected by HAS. This rarely occurs in France as most drugs considered not to provide any additional benefit would receive an ASMR V rating. Another contrast between the recommendations issued based on cost-effectiveness and those based on clinical benefit (HAS) was seen for eltrombopag and imatinib, which received high ratings in France (important SMR and ASMR II–III), but were rejected and restricted by NICE and SMC, respectively. These examples emphasise the magnitude and contradictory nature of these differences. Implications for patients and society are significant in terms of access and efficiency in the use of healthcare resources. Results describe the similarities and differences identified at each stage of the decision process, how they compare across countries and contribute to explaining cross-country differences.

### Evidence

The same primary trials were considered, which were predominantly phase III RCTs for eight of the study drugs. For the two remaining drugs, the primary trials were phase II due to the early marketing authorisation received (e.g. trabectedin, ofatumumab). These primary trials had relatively small sample sizes (e.g. less than 300 participants in 60 % of trials) and decisions often relied on results from subgroup analyses (e.g. 50 % of cases). Comparators were standard care except two cases comparing different doses of the treatment under investigation (e.g. mannitol dry, trabectedin) and one case with no comparator (e.g. ofatumumab). For 80 % of the study drugs, the primary endpoints were surrogate and predominantly validated with the exception of “time-to-progression” for soft tissue sarcoma and “platelet response” for idiopathic thrombocytopenic purpura. In two cases, NICE’s main outcome of interest was “overall survival” despite it not being the trial’s primary endpoint (e.g. imatinib, ofatumumab).

The inclusion of the remaining non-primary trials had very little influence on the assessment. Outcomes from these trials were generally not reported, and when reported, the type of data provided was around safety (e.g. romiplostim, ofatumumab, eltrombopag), dosage research (e.g. eltrombopag) and historical controls (e.g. trabectedin).

Focusing on the economic evidence, similar cost-utility models were considered by NICE, SMC and TLV except for eltrombopag, for which a cost-minimisation analysis was considered by TLV. Additionally, the comparators used by NICE and SMC for eltrombopag were different: NICE considered conventional care, while SMC and TLV considered romiplostim. No cost-effectiveness models were included in the HAS reviews, as cost-effectiveness was not a requirement for first time approvals at the time of the study.

Different evidence was included by some agencies and not by others. When comparing the trials considered by NICE to those considered by SMC, TLV and HAS, 1 out of 19 trials, 4 out of 15, and 6 out of 23, respectively, were not included in the NICE appraisals. These included a database used to estimate HRQol data for trabectedin for SMC; two open-label trials (eltrombopag) and two registries (romiplostim) for TLV; and four phase II open-label trials (azacitidine, eltrombopag), one post-marketing surveillance survey (study extension for eltrombopag) and one indirect comparison (trabectedin) for HAS. HRQol data was not specifically reported in five out of ten cases, and in four other cases, it was not reported homogeneously across the board.

These differences in the evidence appraised were associated with differing HTA outcomes in five cases (Table 2): (a) the inclusion of registry data for trabectedin by NICE as historical controls; (b) different primary endpoints for mifamurtide (“overall survival” for NICE and “progression-free survival” for SMC, TLV and HAS); (c) the secondary endpoint “severe bleeding events” for eltrombopag only reported by NICE; (d) the lack of HRQol data in the assessment of eltrombopag for HAS; and (e) different economic models for eltrombopag.

**Table 2:** Cases where differences at each step of the HTA process explain differences in HTA recommendations

Drug and indication pair		Eltrombopag	Imatinib	Mannitol dry	Mifamurtide	Trabectedin
		Idiopathic thrombocytopenic purpura	Gastro intestinal stromal tumours (adjuvant, after surgery)	Cystic fibrosis	Osteosarcoma	Soft tissue sarcoma
HTA recommendation	Positively appraised (list or restricted)	SMC, TLV, HAS (ASMR II)	SMC, HAS (ASMR III)	NICE, HAS (ASMR V)	NICE, SMC, TLV	NICE, HAS (ASMR V)
	Rejected	NICE	NICE	SMC	HAS	SMC
Evidence	Differences in the level of evidence reported	✖ Severe bleeding events (WHO grade 3–4) (NICE) ✖ Lack of Qol data (HAS) Qol data included for NICE, SMC and TLV ✖ CUA-standard care (NICE) ✔ CUA-romiplostim (SMC) ✔ CMA-romiplostim (TLV)			✔ Progression-free survival = primary endpoint (SMC, TLV, HAS) ✖ Overal survival = primary endpoint (NICE)	✔ Use of registry data as historical controls (NICE)
Interpretation of the evidence	Different interpretation of the same evidence appraised		Short trial duration ✖ NICE, SMC Not raised by HAS	No reduction in hospital days and use of antibiotics ✖ HAS Not raised by SMC, NICE Qol not improved ✖ HAS ✔ NICE Not raised by SMC		
	Different interpretation of the same uncertainty	Short trial duration ✖ NICE (experts), SMC, TLV ✔ HAS (same as comparator)	Overall survival not significantly improved ✖ NICE ✔ SMC (orphan) ✔ HAS (on-going trial)	Risk of bronchospasms ✖ HAS ✔ NICE (expert opinion) Not raised by SMC	Risk of interaction between treatments ✖ HAS (other study) ✔ NICE, SMC (expert opinion), TLV (longer-term data)	Lack of comparative evidence (phase II non-comparative pivotal trial) ✖ HAS ✔ NICE (rarity, early marketing authorisation, historical controls) ✔ SMC (rarity, investigational nature of the treatment)

*NICE* National Institute for Health and Care Excellence, *SMC* Scottish Medicines Consortium, *TLV* Pharmaceutical Benefits Board, *HAS* Haute autorité de Santé

### Interpretation of the evidence

**Fig. 2:**
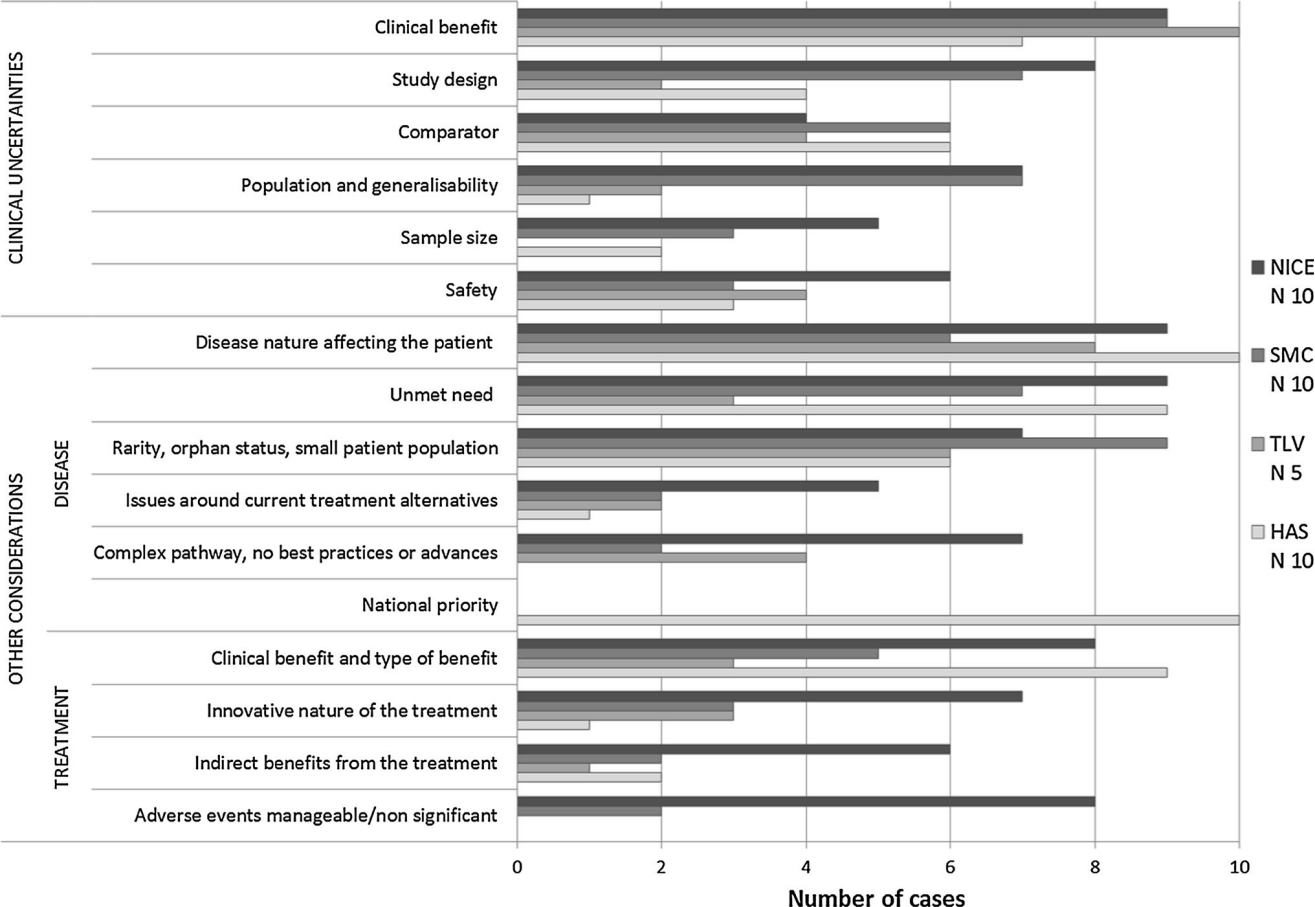
Illustrates the number of cases where clinical uncertainties and “other considerations” were identified influencing the decision process in each country. In total 124 clinical uncertainties were identified across the 35 country drug-indication pairs grouped into ten categories, and 125 “other considerations” grouped into 16 categories. The latter 16 categories were further distinguished between those that relate to living with the disease in question, from those to taking the treatment. The representation of each group was ordered such that the more frequently identified clinical uncertainty, disease-related and treatment-related “other considerations” are represented at the * top* of the * graph*

When appraising the evidence, a number of concerns were raised and recorded in the HTA reports. This “uncertainty” was identified 124 times (*N*_u_) and grouped into ten categories, depending on the type of concern raised (Fig. 2). This interpretative component occurs during the deliberative process, during which these concerns may or may not be considered acceptable based on the decision-makers’ judgments, and, in some instances, on stakeholder input. Their judgment may be influenced by “other considerations” relating to disease and treatment characteristics, which may not be captured in the standard estimates of HTA. In this respect, 125 individual “other considerations” (*N*_oc_) were identified and grouped into 16 categories (Fig. 2).

**Fig. 3:**
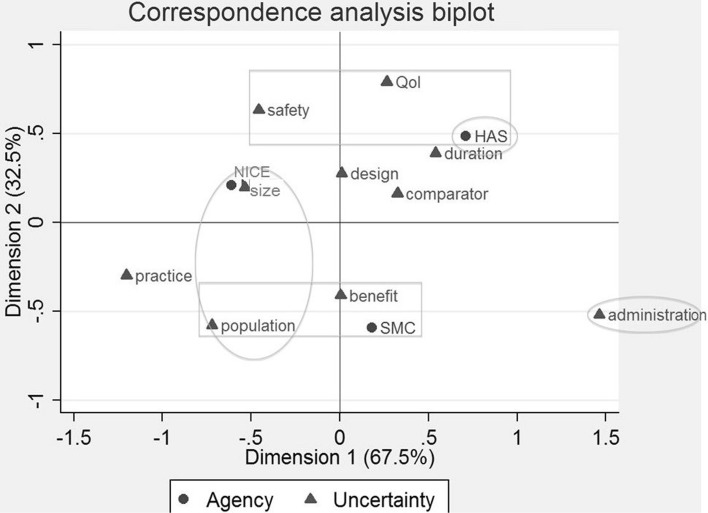
This figure represents the correspondence analysis biplot illustrating the relative associations between the HTA bodies and the clinical uncertainties raised by each HTA body. Although the null hypothesis of independence was rejected (χ^2^ = 18.80; *p* = 0.4040), it provides an indication about specific risk preferences. On the horizontal axis (67.5 % of the variation), NICE is more likely to be concerned about population generalizability and conformity to clinical practice than HAS, which was more concerned about issues relating to the treatment’s administration and provision, and the duration of the trial. On the vertical axis (32.5 % of the variation), SMC was more likely to be concerned about population generalizability and the treatment’s benefit, and HAS about quality of life improvement and safety. Conducting the same analysis across the five study drugs appraised by all agencies, a non-significant association between variables was seen, likely due to the small sample size (χ^2^ = 27.95; *p* = 0.3451). Nevertheless, similar results were seen, with additionally NICE being relatively more likely to be concerned about sample size, HAS with the duration of the study, and TLV about the treatment’s administration and provision. *NICE* National Institute for Health and Care Excellence, *SMC* Scottish Medicines Consortium, *TLV* Dental and Pharmaceutical Benefits Board *HAS* Haute Autorité de Santé, *Qol* quality of life, *safety* safety assessment, *design* trial design, *comparator* comparator, *duration* duration of the trial, *administration* administration and provision of the treatment, *benefit* benefit of the treatment, *size* sample size, *population* population generalizability, *practice* clinical practice

The correspondence analysis biplot illustrates agency-specific risk preferences for these ten drugs, identifying the types of concerns that one agency is more likely to raise compared to another agency (Fig. 3). NICE was relatively more concerned about population generalizability compared to HAS, which was more concerned about issues related to the treatment’s administration and provision (horizontal axis). In contrast, SMC was relatively more concerned about population generalizability and the treatment’s benefit, and HAS about quality of life improvement and safety (vertical axis). Conducting the same analysis across the five study drugs commonly appraised, similar results were seen, with additionally NICE being relatively more likely to be concerned about sample size, HAS with the duration of the study, and TLV about the treatment’s administration and provision.

**Fig. 4:**
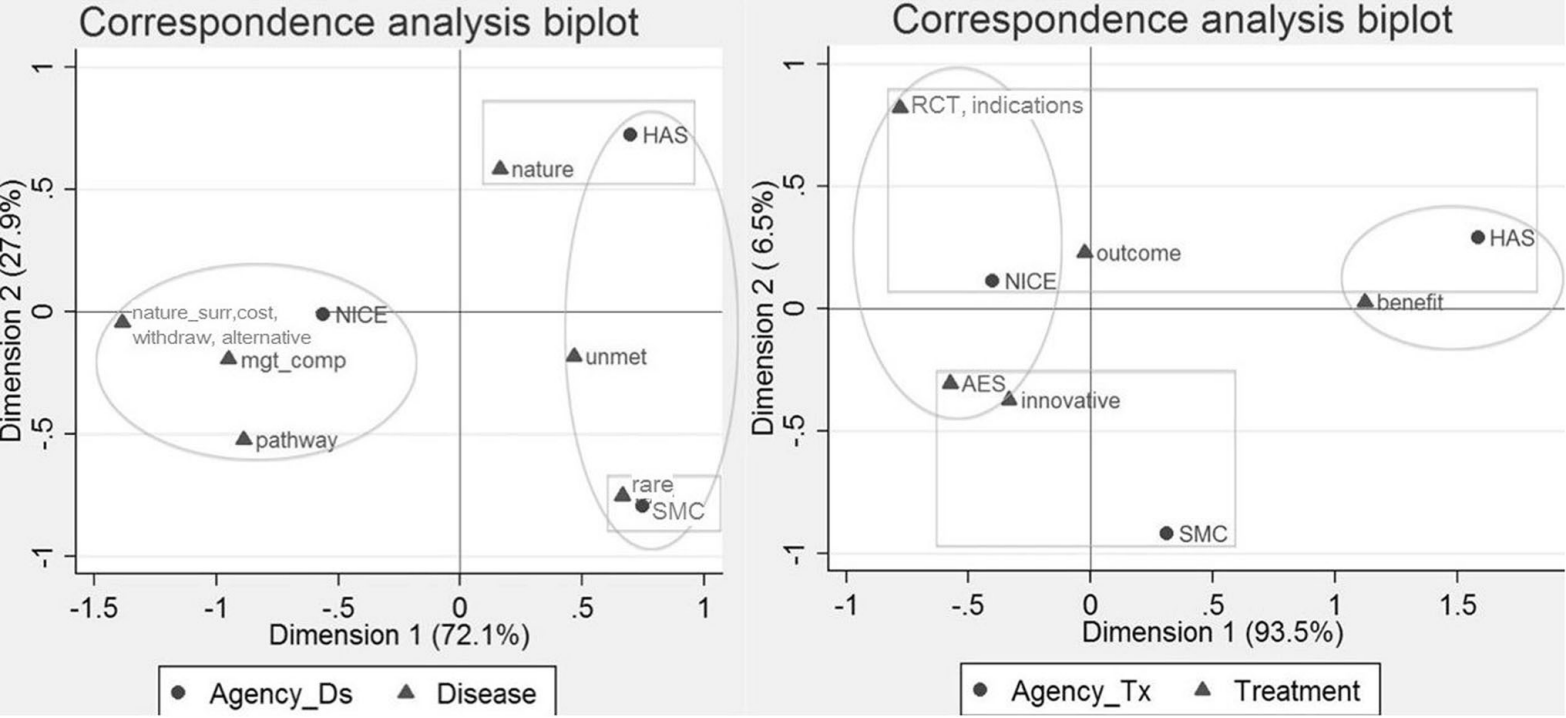
Correspondence analysis biplot illustrating the relative associations between the HTA bodies and disease (*left*) and treatment characteristics (*right*). The figure to the *left* represents the statistically significant relative associations between the HTA bodies and disease characteristics (χ^2^ = 40.05; *p* = 0.0008). On the horizontal axis (72.1 % of the variation), NICE was more likely to account for existing treatment alternatives, clinical practice and the impact of the disease on the patient’s surroundings, whereas SMC and HAS for rarity and unmet need. On the vertical axis (27.9 % of the variation), HAS was more likely to value the nature of the disease, and SMC the rarity of the condition. Conducting the same analysis across the five drugs appraised by all agencies, associations were statistically significant (χ^2^ = 47.37; *p* = 0.0008). Preferences for NICE, SMC and HAS were similar, whereas TLV was relatively more likely to value the nature of the condition (e.g. disease severity). The figure to the *right* illustrates the significant relative associations between the HTA bodies and treatment characteristics (χ^2^ = 29.46; *p* = 0.0011). On the horizontal axis (93.5 % of the variation), NICE was relatively more likely to value the treatment’s safety and challenges in conducting RCTs, and HAS the drug’s clinical benefit compared to other agencies. On the vertical axis, relationships were relatively less meaningful given that only 6.5 % of the variation was captured. Conducting the same analysis across the four drugs appraised by all four agencies, similar conclusions were reached (χ^2^ = 21.05; *p* = 0.0496). Additionally, TLV was relatively more likely to value the innovativeness of the treatment compared to the other agencies. *NICE* National Institute for Health and Care Excellence (NICE), *SMC* Scottish Medicines Consortium, *TLV* Dental and Pharmaceutical Benefits Board, *HAS* Haute Autorité de Santé, *rare* rarity, small sample size, orphan drug, *unmet* unmet need, *nature* nature of the condition and its impact on the patient, *pathway* complex pathway, no best practice, *alternative* issues around current alternatives, *cost* cost burden of current treatment alternatives, *nature* **_** *surr* disease nature affecting the patient’s surroundings, *withdraw* withdrawals from effects not related to the treatment, *mgt* **_** *comp* issues around the management of treatment alternatives, *benefit* clinical benefit and type of benefit, *outcome* indirect benefits from the treatment, *innovative* innovative nature of the treatment, *AES* adverse events from the treatment manageable or non-significant, *RCT* challenges in conducting RCTs, *indications* additional indications of treatment

The same analysis was undertaken focusing on disease and treatment characteristics, to understand the trends in the types of value judgments made across settings and for these ten drugs. Focusing on preferences relating to disease characteristics (Fig. 4), NICE was relatively more likely to account for existing treatment alternatives, clinical practice and the impact of the disease on the patient’s surroundings, whereas SMC and HAS were more likely to value rarity and unmet need (horizontal axis). In contrast, HAS was relatively more likely to value the nature of the disease compared to SMC, which was more likely to value the condition’s rarity (vertical axis). Conducting the same analysis across the five drugs appraised by all agencies, TLV was additionally relatively more likely to value the nature of the condition (e.g. disease severity). Correspondence analysis examining relative value preferences around treatment characteristics across the ten study drugs (Fig. 4) showed that NICE was relatively more likely to value the treatment’s safety and challenges in conducting RCTs, and HAS the drug’s clinical benefit compared to the other agencies. Conducting the same analysis across the five drugs commonly appraised, similar conclusions were reached where additionally TLV was relatively more likely to value the treatment’s innovativeness.

The risk and value preferences identified across the ten study drugs may have influenced these processes and contributed to explaining cross-country differences. Examining each of the concerns more in depth, only 14.5 % of the uncertainties identified (18 of the *N*_u_ = 124) were commonly raised by all agencies, the remainder having been raised by only one or some of the agencies. This was further confirmed when measuring agreement in the clinical uncertainties raised when appraising the same evidence between two agencies, which ranged from poor to less than expected by chance (*κ* range −0.30 to 0.08) (Table 3). In four cases, these differences in interpreting the same evidence related to one of the main reasons for the final decision (Table 2). For imatinib, the primary trial length was deemed too short by NICE and SMC; this was not highlighted by HAS. Additionally, the secondary endpoint “overall survival”, considered by NICE as the main parameter of interest, was not significantly improved, negatively influencing the decision (e.g. imatinib was rejected by NICE). For mannitol dry, the lack of improvement in hospital days and antibiotic use reduction was a concern for HAS, but not for NICE or SMC, further illustrating the impact that agency-specific risk preferences may have on decisions (HAS was relatively more concerned about issues relating to the treatment’s administration and provision compared to the other agencies, Fig. 3). The lack of improvement of HRQol negatively influenced HAS’s decision (ASMR V, in line with findings from Fig. 3), and was also raised by NICE, who acknowledged that current measures do not fully capture the treatment and disease effects; this was not highlighted by SMC.

**Table 3:** Agreement between HTA bodies in the uncertainty raised about the same evidence (raised versus not raised); and when the same uncertainty was raised, agreement about how it was dealt with (addressed versus not addressed)

Kappa scores (95 % confidence intervals) standard error (SE) number of observations (*n*)	Level of agreement in the uncertainties raised (raised versus not raised)				
Level of agreement in interpreting the same uncertainties (addressed versus not addressed)		NICE	SMC	TLV	HAS
	NICE	1	−0.06 (−0.235 to 0.124) SE = 0.091 *n* = 117	−0.15 (−0.434 to 0.143) SE = 0.147 *n* = 44	0.01 (−0.172 to 0.183) SE = 0.090 *n* = 110
	SMC	0.51 (0.203–0.814) SE = 0.156 *n* = 29	1	−0.30 (−0.588 to 0.018) SE = 0.145 *n* = 43	0.08 (−0.108 to 0.261) SE = 0.094 *n* = 110
	TLV	1.00 (1.00–1.00) SE = 0.00 *n* = 7	0.72 (0.232–1.00) SE = 0.249 *n* = 7	1	−0.07 (−0.324 to 0.180) SE = 0.128 *n* = 44
	HAS	−0.08 (−0.227 to 0.067) SE = 0.075 *n* = 24	0.18 (−0.272 to 0.630) SE = 0.230 *n* = 22	−0.50 (−1.00 to 0.235) SE = 0.375 *n* = 4	1

Cohen’s kappa scores (*κ*) rank agreement levels from poor (*κ* = 0) to perfect agreement (*κ* = 1) and where minus values of *κ* correspond to cases when agreement was less than expected by chance

*NICE* National Institute for Health and Care Excellence (NICE), *SMC* Scottish Medicines Consortium, *TLV* Dental and Pharmaceutical Benefits Board, *HAS* Haute Autorité de Santé

Agreement between two agencies was reached if a concern was considered addressed or not by both, and disagreement if addressed by one and not the other. There was agreement for 13 and disagreement for five of the 18 concerns commonly raised. When comparing agreement in how agencies dealt with the same concerns across pairs of countries, it varied, ranging between moderate to lower than expected by chance, depending on the agencies (*κ* range −0.50 to 1.0) (Table 3).

Between 5 and 51 % of these clinical uncertainties (*N*_u_ = 124), depending on the country, were addressed through various means (51 % of *n*_u_^nice^ = 68 uncertainties for NICE; 12 % of *n*_u_^smc^ = 60 for SMC; 47 % of *n*_u_^tlv^ = 21 for TLV; and 5 % of *n*_u_^has^ = 44 for HAS). First, stakeholder input was used to confirm the plausibility of a (uncertain) clinical claim. Second, the uncertainties were raised but nevertheless considered acceptable by the Appraisal Committee. Third, greater uncertainty was accepted given the rarity of the condition or accounting for non-primary evidence. In three cases, differences in the interpretation of evidence were also one of the main reasons for the final recommendation (Table 2). Two of these were based on expert opinion: the risk of bronchospasms was deemed minimal by NICE clinical experts for mannitol dry, and the risk of interactions with other treatments was deemed minimal by clinical experts from NICE and SMC for mifamurtide. In one case (trabectedin), the lack of comparative data for the primary phase II trial was a concern for all but was addressed differently. It was deemed acceptable given the rarity of the condition and investigational nature of the treatment by NICE and SMC; additionally NICE accounted for registry data as historical controls; in contrast, it was not deemed acceptable by HAS.

A number of additional “other considerations” were also put forward by the agencies as one of the reasons for the final recommendation, and associated with differing final outcomes. In a number of cases, greater flexibility was granted to the ICER and uncertainty on the basis of the following considerations that relate to agency-specific modulators: (a) SMC modifiers (5/10 drugs), (b) NICE end-of-life supplementary advice (4/10 drugs) [43], or (c) disease severity at TLV (all five drugs). In particular, four drugs fulfilled the NICE end-of-life criteria, where three were considered cost-effective with an ICER lower than £50,000/QALY (lenalidomide, azacitidine, trabectedin), and one not cost-effective with an ICER greater than £50,000/QALY (everolimus). Similarly, the high ICERs were accepted by SMC for lenalidomide and azacitidine, given the SMC modifiers, and by TLV for lenalidomide, given the severity of the disease.

There were also a number of process-specific modulators rendering the ICER more acceptable, that contributed to explaining cross-country differences: (a) patient access schemes at NICE (7/10 drugs) and SMC (3/10 drugs), (b) lower discount rates accepted by NICE and SMC (1/10 drugs), (c) imposing a restriction by NICE (3/10 drugs) and SMC (4/10 drugs), (d) imposing a re-assessment by TLV (2/5 drugs) and HAS [8/10 under a temporary authorisation scheme (ATU)]. For example, uncertainty was addressed for lenalidomide by imposing a third line restriction (SMC, NICE), or a future re-assessment once more evidence is collected (TLV). Another modulating factor was the ability to implement a lower discount rate on costs and effects captured in the model, as was seen for mifamurtide by NICE and SMC, whereas the high ICER was acceptable for TLV given the severity of the condition, but was rejected by HAS for the reasons discussed in the next paragraph.

A final contrast was seen when assessing cost-effectiveness versus clinical benefit, also resulting in opposite conclusions. A number of compounds rejected by NICE and SMC received an important SMR rating with a 65–100 % coverage rate (e.g. eltrombopag, everolimus, imatinib), and a high ASMR rating associated with a more favourable pricing scheme (e.g. eltrombopag, imatinib). The negative recommendations issued by NICE and SMC were due to the high ICER and main parameter of interest included. There were also drugs positively appraised by NICE and SMC, which received very low SMR ratings [e.g. moderate (30 %) and weak (15 %) coverage] and an ASMR V or a rejection by HAS (e.g. mannitol dry, ofatumumab, mifamurtide). This was because of the lack of comparative data as a result of the early marketing authorisation granted (ofatumumab) and early scientific advice received (mannitol dry), or the highly uncertain evidence presented (mifamurtide). Mannitol dry and mifamurtide also had in common that they were the only two drugs that were not part of the temporary authorisation scheme (ATU) in France.

## Discussion and policy implications

This study adopted a mixed methods research design based on an existing methodological framework to investigate HTA decision processes for ten drug and indication pairs across four countries, and showed important variations and contradictory trends across countries. Differences at each stage of the HTA process were identified, partly explaining the reasons for differing HTA recommendations across countries, while illustrating the complexity of these processes. First, heterogeneity was seen in the evidence accounted for, in the interpretation of the same evidence, and in the different ways of dealing with the same uncertainty (Table 2). These were influenced by the evidentiary, risk and value preferences identified across the ten study drugs. The differences in interpreting the same evidence were partially explained by varying levels and types of stakeholder input, the consideration (or not) of the drug’s orphan status or investigational nature, the consideration of additional qualitative criteria (e.g. innovation, unmet need), the presence of another study, or as part of the decision-maker’s judgment during deliberation. There were also a number of decision modulators that contributed to a greater acceptance of uncertainty or higher and uncertain ICERs. These included agency-specific modulators, pertaining to agency-specific elicited or non-elicited societal preferences, such as the SMC modifiers, NICE’s end-of-life supplementary advice and disease severity for TLV. There were also process-specific modulators, which included the ability to implement patient access schemes or lower discount rates, or to impose restrictions or future re-assessments. There were also consequences from the HTA approach used (clinical or cost-effectiveness) on the final decision.

Results from this in-depth analysis of ten orphan drugs suggest that HTA is not a simple analysis of clinical and/or cost-effectiveness, but remains a flexible process subject to the decision maker’s interpretation about uncertainty and social values as part of the deliberative process of HTA. This study contributes to shedding light on some of the factors being accounted for, which may not necessarily be explicitly defined as part of the decision process. Policymakers should be aware of the more comprehensive set of factors accounted for in these decisions, and the different ways of applying HTA, including how countries dealt with the issues specific to—but not limited to—orphan drugs. The implications of these findings are discussed here, together with the study limitations.

### Contrasting applications of HTA

A first contrast was seen between the HTA recommendations driven by cost-effectiveness and those by clinical benefit. Some drugs with a recognised positive clinical benefit in France were rejected in some, but not all, of the other countries partly due to their high ICER (e.g. everolimus, eltrombopag). This finding is in line with one study that compared NICE coverage and HAS ASMR decisions for a sample of anticancer drugs, showing a significant association between the QALY gain and ASMR ratings, but none when accounting for costs (ICER) [8]. This also has implications on price, which is driven by the ASMR assessment. Economic evaluation has recently been implemented by HAS to support price negotiations for those drugs with an ASMR I-III rating (significant to major improvement in clinical benefit). In such cases, the economic evaluation acts as an additional criterion to be accounted for by the French Economic Committee for Healthcare Products (CEPS) when negotiating prices, giving more weight to the concept of value and value for money. This two-step approach may, however, have negative implications on the price of those orphan drugs considered to have a minor or no improvement in clinical benefit (ASMR IV-V). As illustrated in the case studies analysed, those drugs with very uncertain evidence (due to the lack of comparative data) received low ASMR ratings, where their price will be set lower than comparator prices. In the other study countries, their assessments based on economic evaluation approaches allow for various techniques to deal with uncertainty (e.g. sensitivity analysis), which subsequently may also influence the ICER estimate and drug pricing.

Further contrasts were also seen within those countries assessing cost-effectiveness. The acceptability of the ICER, based on similar economic models and comparators, differed due to the agency-specific or process-specific modulators identified: (a) disease severity for TLV, (b) SMC modifiers, (c) patient access schemes, (d) NICE end-of-life criteria, (e) imposing restrictions, or (f) continuous data generation and future re-assessment. The first four reflect adjusted willingness-to-pay thresholds and special considerations for orphan drugs, while the latter two cases relate to the ability to modulate the ICER by identifying circumstances or subgroups for which the treatment is cost-effective, or accepting greater uncertainty for a limited period of time until more evidence is generated. Findings for Sweden are in line with a recent study that demonstrated the positive impact of disease severity on reimbursement decisions, despite severity not being explicitly defined [44]. The ability to implement patient access schemes is another way of improving the cost-effectiveness and/or uncertainty [45], and providing earlier access to these treatments [46]. Their effects on innovation and expected returns are still unclear [47], and a number of issues around their implementation have been already noted [48]. Additionally, in those countries that have the ability to implement process-specific modulators (e.g. patient access schemes), this study showed that their application was not the same nor consistent across countries or drugs.

### Dealing with rare conditions

Results illustrate the type of issues encountered when dealing with orphan drugs in terms of the nature of the evidence presented (e.g. sample size, phase II primary trials, subgroup data, surrogate endpoints, lack of comparative data) and the types of issues highlighted by the HTA bodies (e.g. small sample size, insufficient statistical power, surrogate endpoints, subgroup data, etc.), corresponding to what characterises orphan drugs [49, 50]. Different ways in dealing with this imperfect evidence were seen. In some cases, these issues relating, but not specific to orphan drugs were considered acceptable through various means as highlighted in this study. This included the specific consideration of the condition’s rarity or the recognised difficulties in recruiting sufficient patient numbers in trials, as highlighted by TLV for eltrombopag or NICE for mifamurtide and romiplostim. In other circumstances (e.g. dealing with subgroup populations), some issues remained inconclusive for all because of their lack of statistical power or retrospective nature (e.g. azacitidine or mannitol). When comparing the prevalence rates used by SMC in their budget impact analysis and the HTA recommendations issued, two observations arise. The three drugs treating less than 20 patients per year (ofatumumab, mifamurtide, trabectedin) had generally poorer outcomes: they all received the poorest ASMR (V) rating, and were more likely to be rejected by the other agencies (ofatumumab by all, trabectedin by SMC). This was a consequence of the lower quality of the evidence from small sample sizes or the lack of comparative data. In the “more prevalent” rare conditions analysed (between 200 and 300 patients per year in Scotland), similar issues were encountered but to a lesser extent were these linked to the small sample size (eltrombopag, mannitol dry). These experiences could be a good starting point for generating the circumstances under which small sample sizes or other issues specific to rare diseases may be acceptable due to the rarity of the condition, also ensuring these are accounted for consistently across cases.

Results also suggest possible misalignments between the incentives implemented for marketing authorisation and their effect at HTA level. For three drugs, the evidence presented was very uncertain due to its low quality and lack of comparative data (e.g. mannitol dry, ofatumumab, trabectedin). This was a consequence of the early marketing authorisation granted or early scientific advice received, which negatively influenced the HTA decisions made: low ASMR ratings (V) in France and rejected in the other countries. Two exceptions, however, were identified (NICE’s recommendations for mannitol dry and trabectedin), where uncertainty was deemed acceptable thanks to the different mechanisms modulating the ICER or to the consideration of other forms of evidence (e.g. historical controls, other considerations). These examples may constitute ways forward in dealing with such scenarios in the future. Additionally in France, all study drugs were made available as part of their temporary authorisation scheme (ATU), with the exception of mannitol dry and mifamurtide. The former received an ASMR V rating and the latter was rejected, which occurs very rarely in France. This may imply that continuous data collection is an additional factor that contributes to accepting greater uncertainty in France.

### HTA methodological challenges

RCT weaknesses are well known and include limitations around safety and generalizability to heterogeneous populations or clinical practice, as well as the cost to conduct them [14]. Similar issues were identified in this study (e.g. generalizability to local population, non-inclusion of certain patient subgroups or subgroup heterogeneity, trial population non-representative of the indication under review, or imbalances in the characteristics or responses across the different subgroups). Given the preference for RCTs observed and the inclusion of these trial results as main parameters of interest in the economic models, the above concerns identified and the diverging ways in dealing with these emphasise the need to recognise complementary forms of robust and valid evidence [14]. Apart from a few cases (e.g. expert opinion to confirm generalizability), this was not seen in practice given the limited role of non-phase III evidence in the assessment of clinical benefit and cost-effectiveness observed in this study. The uptake of such forms of evidence is still modest and likely due to the lack of expertise around dealing with a variety of types of observational evidence including those based on real world data such as electronic patient records, [51] or patient-reported outcomes [52]. Their role, however, is to be stressed given their potential use for policy making in, for example, the value-based system or process for highly specialised medicines at NICE, the patient and clinician engagement (PACE) programme at SMC, the use of managed entry agreements [47] and, more recently, the introduction of a pilot study on adaptive licensing at the EMA [53, 54]. With these new developments, the environment is increasingly relying on expert opinion, observational studies and real world data [55], which could provide insights about treatment effectiveness, the burden of illness, the nature of a condition, or the indirect health care costs and benefits from taking the treatment and feeding it into a more adaptive model of HTA [56]. This is already in place in some countries such as Sweden or France (under the ATU scheme), which has contributed to dealing with uncertainty in some of the cases evaluated without imposing additional conditions or restrictions.

This study identified differences across countries in the type of evidence that is considered appropriate and in interpreting the same evidence, contributing to explaining different HTA recommendations. A more formalised and consistent recognition of the acceptability criteria for evidence and uncertainty is needed, which could be achieved by generating criteria based on past decisions such as the specific circumstances (e.g. early marketing authorisation) or quality standards (e.g. reliability, validity) required. The agency-specific risk and value preferences identified in this study could also be a good starting point for shedding light on the more common circumstances already arising in the different countries.

### Practical implications

This research is in line with the recognised need to better understand pricing and reimbursement systems through cross-country learning and sharing of experiences [57]. It may be useful for European-level initiatives, such as the pilot for a common European HTA (EUnetHTA), as it sheds light on the different applications of HTA and the reasons for differences in the HTA recommendations made, which can feed into discussions when seeking greater consensus across Member States. It may also feed into the new programmes that have since been implemented for orphan drugs (PACE programme at SMC), and for ultra-orphan drugs (NICE’s Highly Specialised Technology (HST) programme, SMC’s ultra-orphan drug decision framework), as well as HAS’s recent requirement for an economic evaluation. These recent developments all have in common (with the exception of the HST programme) that they are add-ons to conventional programmes. Therefore, better understanding of how value is being assessed within these conventional programmes and the reasons for cross-country differences is relevant to identifying issues and potential ways forward for their continuous improvement, while acting as a reference when evaluating these new programmes. This is all the more significant given their recentness, where little is known about their impact.

Results and the systematic approach used may also feed into other forms of research around priority setting. The retrospective identification of the criteria driving previous decisions, applied in this study, is also recognised as one approach to criteria elicitation for multiple criteria decision analysis (MCDA) when used for priority setting [58]. When comparing the criteria identified in this study to those elicited by the EVIDEM project for the purpose of MCDA, similarities were seen. For example, unmet need was categorised as unmet need in efficacy, in safety, in patient-reported outcomes and patient demand [59]. This study identified the different expressions of unmet need, such as: the importance of new treatment options, the lack of (satisfactory) treatment alternatives, alternatives not routinely available, the need to improve therapeutic management, and so forth. Identifying the different expressions of such criteria in practice may feed into defining their attribute levels during the criteria elicitation processes (e.g. MCDA, discreet choice experiments).

A more recent study developed a value proposition based on 19 social value arguments about orphan drug reimbursement decisions, summarised into four value-bearing factors (e.g. disease-related, treatment-related, population-related and socio-economic factors) [60]. Most of these factors were identified in this study (Fig. 2), with the exception of the identifiability of treatment beneficiaries, the impact on the distribution of health, or any of the socio-economic factors. These corroborate the finding’s content validity, and showcase the ability to identify how these factors are expressed in practice. Another example is the second component, “decision-making process”, of the evidence-informed framework developed by Dr Stafinski and colleagues, comprising a list of 7 questions important for resource allocation decisions, and which corresponds to the decision-making processes analysed in this study [61]. This research and the approach used allows one to identify how some of the key questions are expressed in practice during these decision processes, namely those about “information inputs” and “information sources”, “social value judgements” and “deliberations”, which correspond to the “evidence” and “interpretation of the evidence” components, respectively, from the methodological framework applied in this study [4].

### Limitations and need for further research

This research is not without its limitations. First, the data was mainly collected from secondary sources. It would have been preferable to have full information about the submissions (e.g. manufacturer submission), but this was not possible in the current scheme. The information obtained by applying the methodological framework was unavoidably limited by the level of detail provided in the HTA reports and whether the framework captures all aspects of the decision-making process [4]. The information published was assumed to be transparent and reflect the main determinants driving the decisions (transparency directive). The analysis of these published documents was considered to provide sufficient detail and explain how decisions were reached. Additionally, triangulation with other data sources ensured that sufficient detail was captured for each case study [e.g. HTA reports, additional material, and input from HTA experts (Advance-HTA consortium, conferences)]. Results were also presented to and discussed with the HTA bodies, ensuring that the interpretation of the decisions made by the research was accurate. Second, there were sampling issues arising from differences among the four agencies in the way they select topics for their assessments. Despite these differences, a suitable sample was identified. Third, this research focused specifically on orphan drugs, which undergo the same HTA process as drugs for more common conditions. Some of the findings may also be applicable to these more common conditions. One component of the analysis did focus on identifying those challenges that are specific, but not necessarily always unique to, dealing with these rarer conditions, and draw key lessons from these. A final limitation is the relatively small sample size, which does not allow for multivariate regression analysis. However, this research resulted in meaningful outputs derived from a more in-depth and qualitative component showing that differences across countries do matter. A more structured understanding of the possible explanations for differences were derived from the findings, allowing for subsequent more quantitative analyses to focus on certain aspects of the decision-making process across a greater sample. Further research could look at the drivers of these differences across a larger sample of drugs and therapy areas using multivariate regression analysis for a greater generalisation of the results, by extending it to other types of drugs to assess how different agencies assess different drug and disease characteristics. In order to maintain the depth and breadth of the analysis building on the methodological framework used in this study, it is highly recommended to begin by prioritising the qualitative strand to ensure that the depth of the processes are captured and comparable across settings.

## Conclusions

This research contributes to better understanding, in a systematic manner, what is driving these complex decision processes in practice, and why countries make different decisions. It also contributes to identifying those factors beyond the standard clinical and cost-effectiveness tools used in HTA, how they influenced the decision and how they were provided. The implications of this research are all the more important given the shift towards niche markets and personalised medicine, where an increasing number of the treatments undergoing regulatory and coverage processes are characterised by some of the important issues discussed in this paper.
